# *Scytosiphon lomentaria* Extract Ameliorates Obesity and Modulates Gut Microbiota in High-Fat-Diet-Fed Mice

**DOI:** 10.3390/nu15040815

**Published:** 2023-02-05

**Authors:** Jing Yan, Jinwoo Bak, Yula Go, Jumin Park, Minkyoung Park, Hae-Jeung Lee, Hyemee Kim

**Affiliations:** 1 Department of Food Science and Nutrition & Kimchi Research Institute, Pusan National University, Busan 46241, Republic of Korea; 2 Department of Food and Nutrition, Gachon University, Seongnam-si 13120, Republic of Korea

**Keywords:** *Scytosiphon lomentaria*, mice, anti-obesity, gut microbiota

## Abstract

*Scytosiphon lomentaria* (SL) is a brown seaweed with antioxidant and anti-inflammatory properties; however, its effects on obesity are unknown. In this research, we investigated the anti-obesity properties and underlying mechanisms of the SL extract in vitro and in vivo. In 3T3-L1 preadipocytes, SL extract inhibited lipid accumulation, decreased the expression of *Acc1*, *C/ebpa*, *Pparg* mRNA and p-ACC1, and increased the expression of *Ucp1* mRNA, UCP1 and p-AMPK. In animal experiments, mice were fed a chow diet, a high-fat diet (HF; 60% of calories as fat), and high-fat diet with SL extract (150 and 300 mg/kg body weight) for eight weeks (*n* = 10/group). SL extract reduced HF-induced weight gain, epididymal fat weight, fat cell size, LDL-C, leptin, fasting glucose, and glucose tolerance. In addition, SL extract had comparable effects on mRNA expression in WAT and liver to those observed in vitro, thereby inhibiting p-ACC1/ACC1 and increasing p-AMPK/AMPK and UCP1 expression. Furthermore, SL extract decreased HF-induced Firmicutes/Bacteroidetes ratio and reversed HF-reduced *Bacteroides* spp., *Bacteroides vulgatus*, and *Faecalibacterium prausnitzii*. These findings suggest that SL extract can aid in weight loss in mice fed a high-fat diet by altering adipogenic and thermogenic pathways, as well as gut microbiota composition.

## 1. Introduction

Obesity is a chronic condition with energy imbalance and adipose tissue inflammation [1]. According to the World Obesity Atlas 2022, more than one billion people will be obese by 2030 [2]. Obesity raises the likelihood of metabolic syndrome, which is linked to dysglycemia, cardiovascular disease, type 2 diabetes, dementia, and cancer [3]. Some anti-obesity drugs can cause side effects, such as diarrhea, fat leakage, flatulence, and fecal incontinence [4,5]. Consequently, it is crucial to investigate natural bioactive compounds that are both safe and effective in preventing and treating obesity. In recent years, phytochemicals from tea, mulberry, wolfberry, and eggplant have been reported to prevent and improve obesity in a safe and efficient manner [6].

It has been reported that seaweeds, particularly brown seaweeds, have anti-diabetic, anti-hypertensive, antioxidant, and anti-inflammatory properties, and they are associated with reduced hyperglycemia, cardiovascular disease mortality, and increased life expectancy [7,8]. Seaweeds are abundant in bioactive compounds, such as polysaccharides (fucoidans), proteins (phycobiliproteins), polyphenols (phlorotannins), n-3 polyunsaturated fatty acids, and carotenoids (fucoxanthin) [9,10,11]. *Scytosiphon lomentaria* (SL) is a brown coastal alga with anti-inflammatory, antioxidant, and anti-cancer properties [12,13]. In previous experiments, SL components were extracted with a variety of solvents and then evaluated for their antioxidant properties. The hot-water extract contained more polyphenols and exhibited a greater DPPH radical-scavenging effect, whereas the ethanol extract had greater hydroxyl radical scavenging activity and anti-lipid peroxidation effect than other solvent-isolated extracts [12,14]. In RAW264.7 cells treated with LPS, SL ethanol extract exhibited anti-inflammatory activities via suppression of NF-κB, resulting in decreased expression of iNOS, COX-2, and IL-1β mRNA [15]. Until now, however, most researches have focused on the anti-inflammatory properties of fucoidan from SL, a specific polysaccharide found in brown algae. Fucoidan derived from SL prevented colon damage in mice lacking dietary fibers by inhibiting oxidative stress and suppressing proinflammatory cytokines by inhibiting NF-κB [16]. Regarding the potential for anti-obesity efficacy, SL extract was observed to reduce metabolic syndrome in obese fish by regulating blood glucose [17]. All the aforementioned in vitro studies indicate that SL extract has the potential to be used as a natural inhibitor and dietary supplement to effectively treat metabolic syndrome; however, in vivo studies have not yet been conducted to identify the anti-obesity effects and underlying mechanisms of the phenolic mixture of SL and its effects on the intestinal flora.

Multiple pathophysiological mechanisms contribute to the development of obesity and metabolic syndromes. Adipogenesis increases cell number by inducing C/EBPα and PPARγ to promote the differentiation of preadipocytes [18], whereas lipogenesis increases fat cell size by inducing ACC1, FAS, and SREBP-1C to promote fatty acid synthesis [19]. Moreover, recent studies have shown that increasing energy expenditure via thermogenesis involving UCP1 and AMPK is a promising strategy for combating obesity [20]. Consequently, the regulation of white fat browning and lipid metabolism, including adipogenesis and lipogenesis, has emerged as a potential anti-obesity target [21] and is being studied as a mechanism underlying the anti-obesity efficacy of numerous natural bioactive compounds.

In addition, gut bacterial composition has been reported to be linked to obesity through modulation of energy production, storage, and consumption [22,23]. Western and high-fat diets have been shown to cause gut microbiota dysbiosis by reducing the diversity of gut microbial composition and increasing the ratio of Firmicutes to Bacteroidetes in humans and obese mouse models [24]. Gut microbial imbalances may lead to metabolic changes and an increase in central appetite, resulting in weight gain [25]. Therefore, enhancing the beneficial gut microbiota is regarded as an important potential target for obesity treatment. However, relatively few experiments have been conducted on *S. lomentaria* polyphenols, and their effects on gut microbiota have not been investigated.

The mechanisms underlying the effects of SL extract on 3T3-L1 cells and animal models of obesity caused by a high-fat diet were explored in this study. We hypothesized that SL extract reduces obesity by suppressing adipogenesis and lipogenesis, enhancing thermogenesis, and modulating gut microbiota.

## 2. Materials and Methods

### 2.1. Preparation of S. lomentaria Extract and LC-MS Analysis

The 70% ethanol-extracted SL extract was obtained from Marine Biobank (Seocheon-si, Republic of Korea) or was isolated from dried *S. lomentaria* purchased from ParaJeju (Jeju, Republic of Korea). The levels of total phenolics and flavonoids were measured using the Folin-Ciocalteu [26] and the diethylene glycol colorimetric assays, respectively [27]. The samples were injected into an Agilent 6530 ESI-Q-TOF-MS (Agilent, Santa Clara, CA, USA) equipped with a 1.7 μm Acquity BEH C18 (2.1 × 50 mm). The mass spectrometer was in the mode of positive ionization. With solvent A (0.1% formic acid (FA) in water) and solvent B (0.1% FA in acetonitrile), the gradient elution was performed as follows: (0–2 min, 98% A and 2% B; 2–20 min, gradual change to 100% B; 20–25 min, 0% A and 100% B; 25–25 min, gradual change to 98% A and 2% B; 26–30 min, 98% A and 2% B; Total 30 min). The parameters were as follows: 9 L/min of gas at 300 °C; 11 L/min of sheath at 350 °C; and 45 psig of nebulizer pressure. The following scan source parameters were used: 4000 V, VCap, and 175 V, Fragmentor. The same method was used to inject quercetin as the standard for the calculation [28].

### 2.2. Cell Viability Assay

3T3-L1 cells were supplied by the Korea Cell Line Bank (Seoul, Republic of Korea). The cells were cultured at 37 °C and 5% CO_2_ in DMEM with 10% FBS, 2.5% HEPES, and 1% penicillin/streptomycin (All from Hyclone, Logan, UT, USA). The cell viability was quantified by Cell Titer Solution (MTS, Promega, Madison, WI, USA). The cells were seeded at 5000 cells/well (96-well), and next day, the cells were treated with SL extracts (0–100 mg/L) for 48 h. The MTS solution was then added for 2 h, and the absorbance was measured using a microplate reader at 490 nm (Multiskan™ GO, Thermo Fisher, Waltham, MA, USA) [28].

### 2.3. Cell Differentiation and Oil Red O Staining

3T3-L1 cells were cultured until they reached 100% cell confluency. Then, the cells were differentiated for 2 days in DMEM containing 10 µg/mL insulin, 0.5 mM IBMX, and 1 µM dexamethasone (All from Sigma, St. Louis, MO, USA) (Days 0–2). The cells were then cultured in DMEM containing 10 µg/mL insulin (Days 3–6). The SL extract (0–20 mg/L) was added to the medium throughout the 6 days of cell differentiation period [28]. Oil Red O (ORO) assay was used to determine the lipid content in the differentiated 3T3-L1 cells [29]. Preadipocytes were seeded at a density of 5000 cells/well in 96-well plates and differentiated using the method described above. At the end of the treatment (Day 7), cells were fixed, ORO solution was added, and microscopy images were taken (JP/BX-FLA; Olympus, Japan). To quantify lipid content, the relative ORO concentration was determined at 500 nm [28].

### 2.4. Animal Studies

The animal experimental design was approved by the Ethics Committee of PNU (Pusan National University; PNU20220130). Forty C57BL/6J mice (male), aged six weeks and weighing approximately 18–22 g, were supplied by Oriental Bio Co. (Seongnam-si, Republic of Korea). After acclimating for one week, mice were randomly separated into four groups: (1) chow diet (Con), (2) high-fat diet (HF), (3) HF with low-dose SL (150 mg/kg; HF-SLL), and (4) HF with high-dose SL (300 mg/kg; HF-SLH). The Con group was fed multigrain diet (Orient, 5L79) for eight weeks, whereas the HF and experimental SL groups were administered an HF (60 kcal% fat, D12492, Research Diets, New Brunswick, NJ, USA) [28]. For eight weeks, the Con and HF groups received 100 μL of PBS orally gavaged daily, while the SL extract groups received 150 mg/kg body weight (bw; HF-SLL) or 300 mg/kg bw (HF-SLH) of SL extract. Body weight and food intake were measured weekly. One day before the mice were killed, a glucose tolerance test (OGTT) was performed. After fasting overnight, the mice were given 2 g of glucose/kg bw orally. One drop of blood from the tail was collected at 0, 30, 60, and 120 min, and the Accu-Chek Instant Meter was used to determine the blood glucose levels (Roche Diabetes Care, Mannheim, Germany). After euthanizing, liver and fat were weighed, and organs, blood, and feces were frozen at −80 °C [28].

### 2.5. Histolopathology of Adipose Tissues and Liver

Adipose tissue and liver were preserved in 4% formalin for 24 h and embedded in paraffin. After staining the sections with hematoxylin and eosin (H&E) and ORO reagents, four images were acquired of each stained slide using an Olympus microscope (JP/BXFLA). ImageJ was used to compute the area of fat cells (NIH, Bethesda, MD, USA) [28].

### 2.6. Blood Analysis

Total triglyceride (TG), cholesterol (TC), and HDL-C levels in the serum were measured using kits (Asan Pharmaceutical Co. Ltd., Seoul, Republic of Korea). Total cholesterol levels were subtracted from HDL-C levels to determine LDL-C concentrations. Serum levels of leptin and adiponectin were detected using a kit (R&D, Minneapolis, MN, USA) [28].

### 2.7. RT-PCR Analysis

RNA was extracted from differentiated 3T3-L1 cells, mice liver, and epididymal tissues of the mice using the RNeasy Mini Kit (QIAGEN, Hilden, Germany) and TRIzol (Thermo Fisher). Total RNA was reverse-transcribed to generate cDNA, which was synthesized with SYBR Green (Bio-Rad) using a CFX real-time instrument (Bio-Rad, Hercules, CA, USA) for RT-qPCR. β-actin was used as the reference. The primers used are listed in Supplemental Table S1 (Macrogen, Seoul, Republic of Korea).

### 2.8. Bacterial DNA Extraction, qPCR, and 16S rRNA Gene Sequencing

Bacterial DNA was isolated from feces using the PowerFecal Pro DNA Kit (QIAGEN). DNA from two mice was combined in equivalent proportions (*n* = 3–4/group) for 16S rRNA sequencing. The samples were purified and sequenced using the iSeq platform (Illumina, San Diego, CA, USA). Trimmomatic (ver. 0.39) was used to clean the sequences, and then the data were organized into operational taxonomic units and aligned with QIIME2 (version 1.9.5) (accessed on 15 December 2022) [30]. In addition, α- and β-diversity analyses were conducted using QIIME2. Quantitative PCR was used to validate the results of 16S rRNA sequencing and to quantify the abundance of 10 well-known probiotics in fecal bacteria (CFX System, Bio-Rad). Bacterial DNA was amplified using the specific bacterial primers listed in Supplemental Table S2 (Macrogen). The ratio of total bacteria was used to calculate the relative abundance (F341/R518) (*n* = 10 per group) [31].

### 2.9. Western Blot

Protein was extracted from the liver tissue and 3T3-L1 cells using M-PER and T-PER reagents (Thermo Fisher) with a protease inhibitor (Thermo Fisher) [28]. After homogenizing, the supernatant was recovered by centrifuging at 14,000× *g* for 5 min at 4 °C. Then, 10 μg of cell protein and 50 μg of protein from mouse liver were loaded into SDS-PAGE and transferred onto membranes. Membranes were first treated in a blocking solution (5% nonfat milk), then with primary antibodies UCP1, AMPK, p-AMPK, p-ACC1, ACC1 (All 1:2000 dilution), and β-actin (1:5000 dilution; All from Cell Signaling, Danvers, MA, USA) at 4 °C overnight. After incubating with a secondary antibody, band intensities were calculated by utilizing a camera (Davinch K, Seoul, Republic of Korea) and ImageJ software [31].

### 2.10. Statistical Analysis

GraphPad Prism 9 (La Jolla, CA, USA) was used for the statistical analysis. The findings are reported as the mean ± SD. One-way ANOVA (Dunnett’s test) or Student’s *t*-test was used to compute the *p*-values, and two-way ANOVA (Dunnett’s test) was performed to examine the effects of treatment and time on the change in mouse body weight. The non-parametric Kruskal–Wallis test was used for calculating the prevalence of certain gut bacteria. The significance level was established at *p* < 0.05.

## 3. Results

### 3.1. Identification of Phytochemical Compounds in S. lomentaria Extract

**Figure 1:**
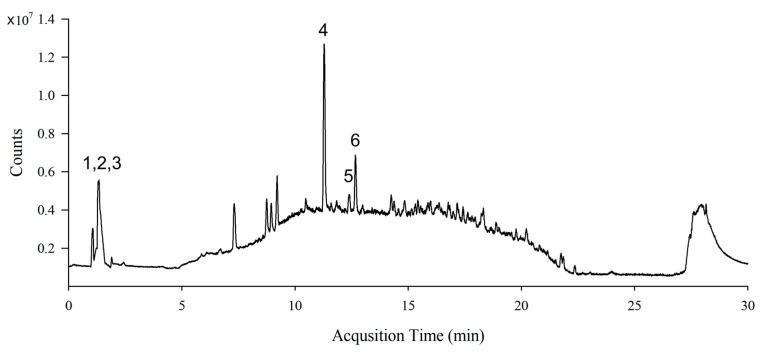
Representative LC-MS chromatogram (total ion current (TIC) plot) of *S. lomentaria* extract. 1. Sinapic acid, 2. Catechin, 3. Quercetin, 4. Hydroxytrifuhalol A, 5. Acacetin, and 6. Caffeic acid.

The total polyphenol content of the SL extract was 13.17 ± 0.48 mg GAE/g extract and the total flavonoid content was 23.75 ± 3.12 mg CE/g extract. LC-MS analysis of the SL extract identified active compounds, such as sinapic acid, catechins, quercetin, hydroxytrifuhalol A, acacetin, and caffeic acid (Figure 1 and Table 1).

### 3.2. Effects of S. lomentaria Extract on the Differentiation of 3T3-L1 Cells

**Figure 2:**
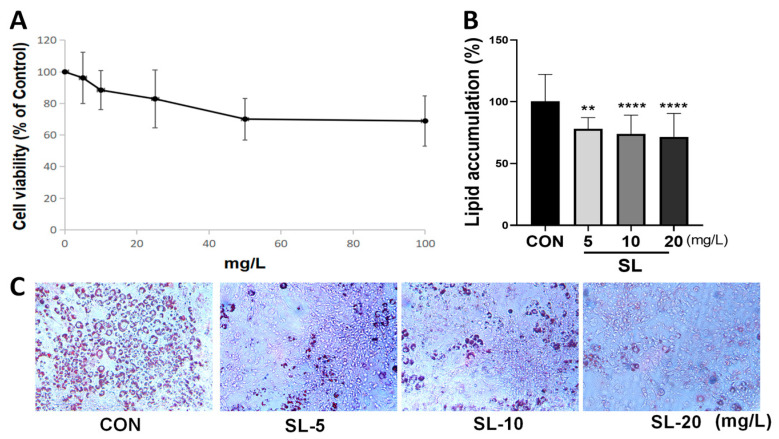
Effects of *S. lomentaria* (SL) extract on cell viability and Oil-Red O (ORO) staining. (**A**) The cell viability was determined after 48 h of incubation with 0–100 mg/L of SL extracts. (**B**) After differentiation with the presence of SL extracts (0–20 mg/L) for 6 days, extracted ORO was measured for concentration at 500 nm. (**C**) ORO staining. Results are expressed as mean ± SD (*n* = 8). Significance was determined as ** *p* < 0.01, **** *p* < 0.0001 using one-way ANOVA (Post hoc Dunnett’s test).

When we treated 3T3-L1 cells with the SL extract at concentrations ranging between 0 and 100 mg/L, their cell viability was higher than 80% at concentrations lower than 20 mg/L (Figure 2A). Concentrations of 10 and 20 mg/L of SL extract were used in subsequent experiments. The SL extract at a concentration of 5 mg/L inhibited lipid droplet formation using ORO stain. Treatment with SL extract at 10 and 20 mg/L significantly decreased lipid droplet accumulation by 73.98% and 71.60%, respectively (Figure 2B,C). The results demonstrated that the SL extract inhibited lipid formation in 3T3-L1 adipocytes without cytotoxicity.

### 3.3. Effects of S. lomentaria Extract on Gene and Protein Expressions Related to Lipogenesis, Adipogenesis, and Thermogenesis in 3T3-L1 Cells

**Figure 3:**
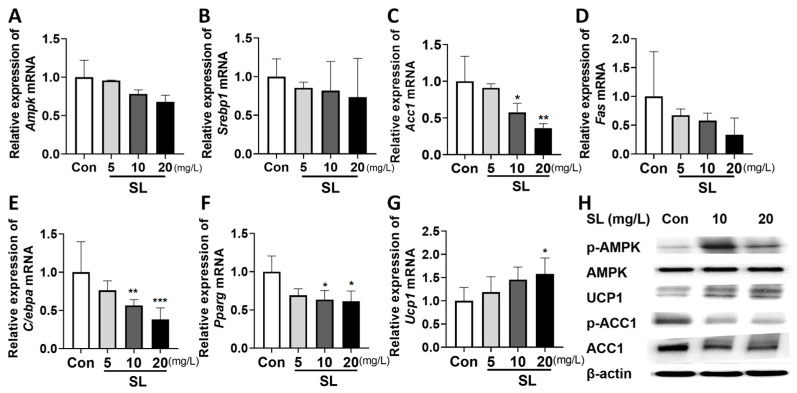
Effects of *S. lomentaria* (SL) extract on gene and protein related to lipogenesis, adipogenesis, and thermogenesis in differentiated 3T3-L1 cells. (**A**) *Ampk*. (**B**) *Srebp1*. (**C**) *Acc1*. (**D**) *Fas.* (**E**) *C/ebpa*. (**F**) *Pparg.* (**G**) *Ucp1*. (**H**) Representative Western blot results. SL extracts induced differentiation in 3T3-L1 cells (0–20 mg/L) for 6 days, and then RNA and protein were isolated. β-actin was used as a control in PCR and Western blot. Results are expressed as mean ± SD (*n* = 3). Significance was determined as * *p* < 0.05, ** *p* < 0.01, *** *p* < 0.001 using one-way ANOVA (post hoc Dunnett’s test).

We determined the underlying mechanisms by which the SL extract inhibited lipid formation (Figure 3A–G). SL extract at 10 and 20 mg/L reduced the expression of lipogenesis-related gene *Acc1* by 63% and 40%, respectively (Figure 3C), and reduced the expression of adipogenesis-related genes *C/ebpa* by 74% and 50% (Figure 3E) and *Pparg* by 70% and 67% (Figure 3F), compared to the PBS-treated control. Moreover, SL extract at 10 and 20 mg/L increased thermogenic gene *Ucp1* by up to 123% and 133%, respectively (Figure 3G), compared to the PBS-treated control. We further measured the protein expression of lipogenic and thermogenic factors, such as p-ACC1, ACC1, UCP1, AMPK, and p-AMPK. When 3T3-L1 cells were treated with the SL extract, the ratio of p-ACC1/ACC1 protein expression was reduced, and UCP1 protein expression and the ratio of p-AMPK/AMPK protein expression were increased compared to the PBS-treated control group (Figure 3H).

### 3.4. Effects of S. lomentaria Extract on Body Weight and Organ Weight in High-Fat-Fed Mice

**Figure 4:**
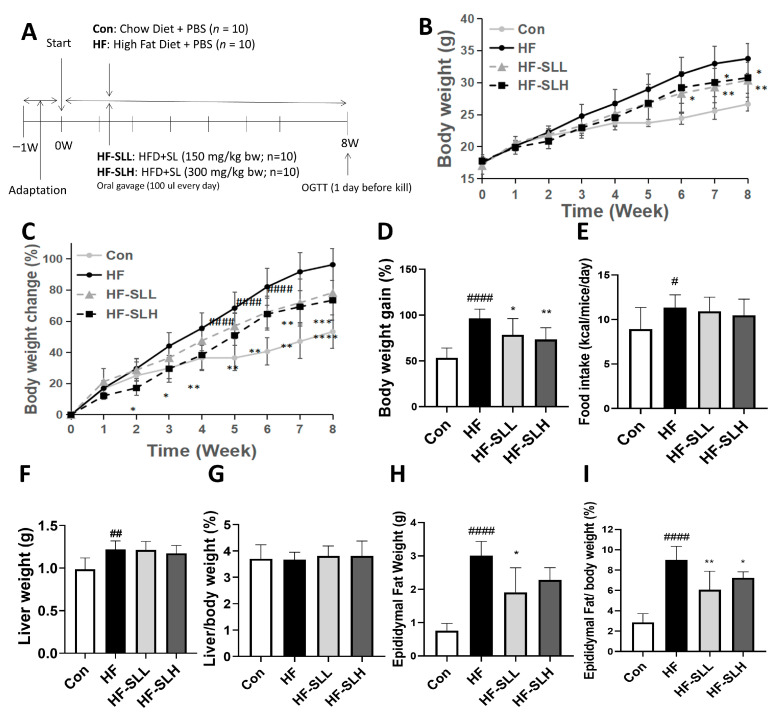
Effects of *S. lomentaria* (SL) extract on body weight and organ weights of mice fed a high-fat diet. (**A**) Timeline for the experiment. (**B**) Weight change trend for 8 weeks. (**C**) Change in body weight calculated by % of body weight each week compared to body weight at Week 0. Compared between the Con and HF group; # *p* < 0.05, ## *p* < 0.01, #### *p* < 0.0001, and compared with the HF group, * *p* < 0.05, ** *p* < 0.01, *** *p* < 0.001, **** *p* < 0.0001 as determined by two-way ANOVA followed by Dunnett’s test (Treatment × Time). (**D**) Body weight gain (%) calculated by (body weight at Week 8—body weight at Week 0)/body weight at Week 0 × 100. (**E**) Average food intake for 8 weeks. (**F**) Liver weight. (**G**) Liver/body weight (at Week 8) ratio. (**H**) Epididymal fat weight. (**I**) Ratio of epididymal fat/body weight (at Week 8). The four diet groups: chow diet (Con), high-fat diet (HF), HF plus SL 150 mg/kg bw (HF-SLL), HF plus SL 300 mg/kg bw (HF-SLH). Data are expressed as mean ± standard deviation (*n* = 10). Compared between the Con and HF group; # *p* < 0.05, ## *p* < 0.01, #### *p* < 0.0001 determined using Student’s *t*-test. Compared with the HF group, significance was determined as * *p* < 0.05, ** *p* < 0.01 using one-way ANOVA (Post hoc Dunnett’s test).

To confirm the results of the in vitro experiment in vivo, mice were fed a high-fat diet to induce obesity and given doses of 150 and 300 mg/kg of SL extract for eight weeks (Figure 4A). Mice in the HF group acquired more weight than mice in the Con group starting at Week 4 (*p* < 0.05), and the trend continued until Week 8. Compared to the HF group, SL extract consumption decreased body weight beginning at Week 6 in the HF-SLH group and at Week 7 in the HF-SLL group (Figure 4B). When the body weight was normalized to the body weight at Week 0, the body weight increased in the HF group starting at Week 3 compared to the Con group. SL extract intake decreased body weight change earlier from Week 2 in the HF-SLH group and at Week 6 in the HF-SLL group. The decline remained significant until Week 8 (Figure 4C). When we compared the body weight gain at the end of Week 8, the HF group gained significantly more weight compared to the Con group, while the SL treatment significantly decreased the body weight gain (Figure 4D). There was no significant variation in dietary intake between the mouse groups, as shown in Figure 4E. Compared to the Con group, liver weight increased significantly in the HF group, but the effect of SL extract on liver weight was not significantly different between groups (Figure 4F). In addition, when liver weight was divided by body weight at Week 8, there was no difference between groups (Figure 4G). As shown in Figure 4H, the weight of epididymal fat increased significantly in the HF group compared to the Con group, but decreased significantly in the HF-SLL group. When calculated by dividing epididymal weight by body weight at Week 8, both SL groups demonstrated a significant reduction in the ratio of epididymal fat/body weight in comparison to the HF group (Figure 4I). It was concluded that SL extract can reduce body fat and weight while maintaining the same caloric intake.

### 3.5. Effects of S. lomentaria Extract on the Adipose Tissue and Liver Histology in High-Fat-Fed Mice

**Figure 5:**
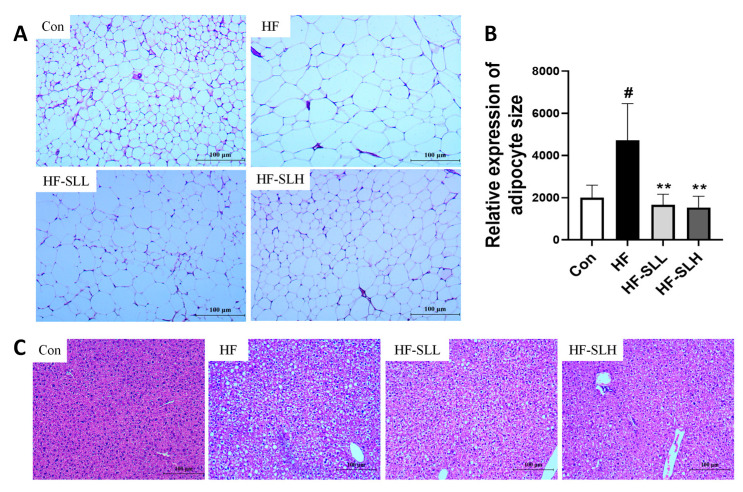
Changes in the histology of WAT and liver in mice fed a high-fat diet. (**A**) A WAT section stained with H&E (×100). (**B**) Relative expression of adipocyte size. (**C**) Section of liver stained with H&E (×100). The four diet groups: chow diet (Con), high-fat diet (HF), HF plus SL 150 mg/kg bw (HF-SLL), HF plus SL 300 mg/kg bw (HF-SLH). Data are expressed as mean ± standard deviation (*n* = 10). Compared between the Con and HF group; significance was determined as # *p* < 0.05 using Student’s *t*-test. Compared with the HF group, significance was determined as ** *p* < 0.01 using one-way ANOVA (Post hoc Dunnett’s test).

Adipocyte size was measured using H&E-stained adipose tissue. As depicted in Figure 5A,B, the area of white adipose tissue (WAT) in the HF group was twice as large as that in the Con group, whereas the size of fat cells was reduced by 65% and 68% following treatment with low and high dosages of SL extract compared to the HF group. The HF group had more spherical hepatocytes than the Con group; however, the number of spherical hepatocytes in the SL extract group was lower than that in the HF group (Figure 5C). ORO staining was conducted to measure intrahepatic lipid droplet; however, no significant accumulation of lipid droplets was observed in the liver.

### 3.6. Effects of S. lomentaria Extract on Serum Profiles

**Figure 6:**
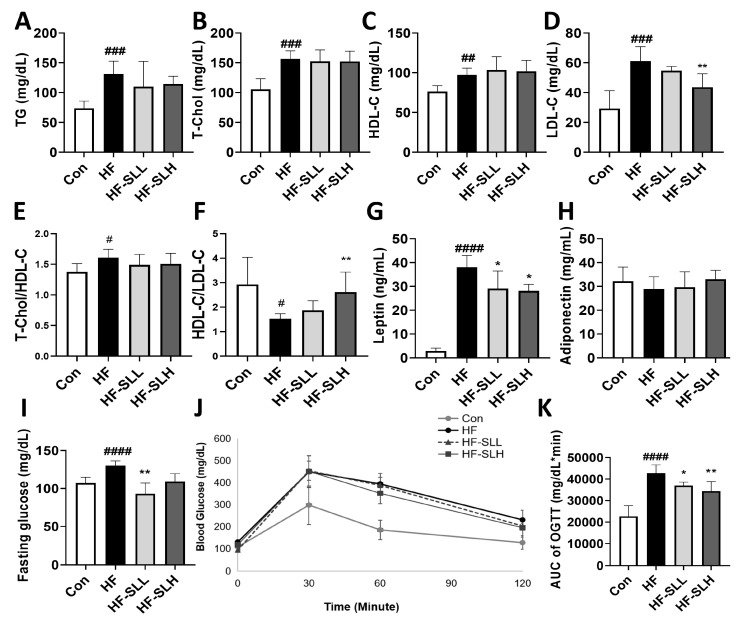
Effects of *S. lomentaria* (SL) extract on the lipid profiles and blood glucose in mice fed a high-fat diet. (**A**) Triacylglycerol (TG). (**B**) Total cholesterol (TC). (**C**) HDL-C. (**D**) LDL-C. (**E**) Ratio of TC to HDL-cholesterol. (**F**) HDL/LDL ratio. (**G**) Leptin. (**H**) Adiponectin. (**I**) Fasting glucose level. (**J**) Curve of OGTT. (**K**) AUC of OGTT. The four diet groups: chow diet (Con), high-fat diet (HF), HF plus SL 150 mg/kg bw (HF-SLL), HF plus SL 300 mg/kg bw (HF-SLH). Data are expressed as mean ± standard deviation (*n* = 10). Compared between the Con and HF group; significance was determined as # *p* < 0.05, ## *p* < 0.01, ### *p* < 0.001, #### *p* < 0.0001 using Student’s *t*-test. Compared with the HF group, significance was determined as * *p* < 0.05, ** *p* < 0.01 using one-way ANOVA (Post hoc Dunnett’s test).

The levels of TG, T-Chol, HDL-C, and LDL-C were significantly higher in the HF group compared to those in the Con group (Figure 6A–D); however, treatment with a high concentration of SL extract (300 mg/kg bw) decreased LDL cholesterol by 29% (Figure 6D). When the clinically utilized indicators were calculated, the T-chol/HDL ratio was, likewise, significantly raised in the HF group; however, there was no significant decrease in the HF-SL groups (Figure 6E). The ratio of HDL to LDL was significantly lower in the HF group compared to the Con group; however, it was significantly higher in the HF-SLH group compared to the HF group (Figure 6F). Leptin and adiponectin are associated with appetite and obesity. As shown in Figure 6G, low and high doses of SL treatment reduced leptin levels by 23% and 26%, respectively; however, adiponectin levels did not vary significantly between SL-treated groups (Figure 6H). Moreover, as shown in Figure 6I, the fasting blood glucose level was increased in the HF group compared to that in the Con group; however, it was decreased in the treatment group with low-concentration SL extract (150 mg/kg bw). The AUC of glucose concentration (OGTT) increased in the HF group but decreased in the treatment groups with high and low doses of SL extract (Figure 6J,K). Compared to the HF group, SL extract significantly reduced hyperlipidemia and hyperglycemia.

### 3.7. Effects of S. lomentaria Extract on the Gene and Protein Expression Related to Lipogenesis, Adipogenesis, and Thermogenesis in WAT and Liver in High-Fat-Fed Mice

**Figure 7:**
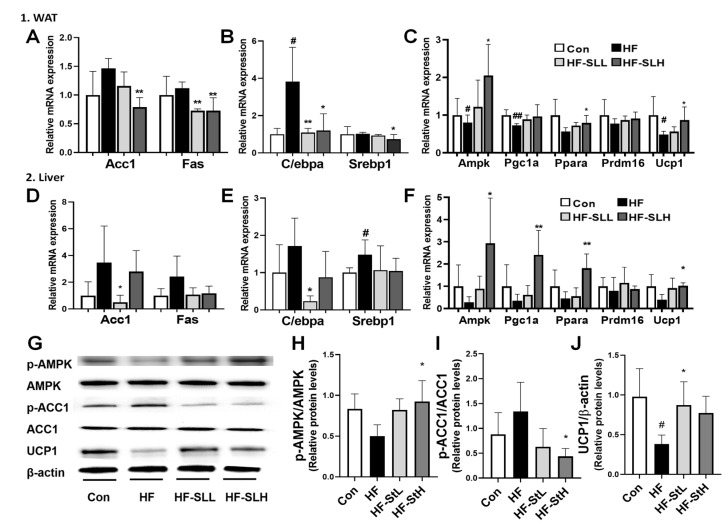
Effects of *S. lomentaria* (SL) extract on the gene and protein expressions related to lipogenesis, adipogenesis, and thermogenesis in WAT and liver. (**A**) *Acc1* and *Fas*, (**B**) *C/ebpa* and *Srebp1*, (**C**) *Ampk*, *Pgc1a*, *Ppara*, *Prdm16*, and *Ucp1* mRNA expressions in WAT. (**D**) *Acc1* and *Fas*, (**E**) *C/ebpa* and *Srebp1*, (**F**) *Ampk*, *Pgc1a*, *Ppara*, *Prdm16*, and *Ucp1* mRNA expressions in liver. (**G**) Western blot in liver. (**H**) Ratio of p-AMPK/AMPK, (**I**) p-ACC1/ACC1, (**J**) UCP1/β-actin. The four diet groups: chow diet (Con), high-fat diet (HF), HF plus SL 150 mg/kg bw (HF-SLL), HF plus SL 300 mg/kg bw (HF-SLH). Data are expressed as mean ± standard deviation (*n* = 10). Compared with the HF group, significance was determined as # *p* < 0.05, ## *p* < 0.01 using Student’s *t*-test. Compared with the HF group, significance was determined as * *p* < 0.05, ** *p* < 0.01 using one-way ANOVA (Post hoc Dunnett’s test).

To clarify the underlying mechanism of SL extract on obesity, the expression levels of genes involved in lipogenesis, adipogenesis, and thermogenesis in WAT and liver were analyzed. As shown in Figure 7A, the high concentration of SL extract reduced the lipogenesis-related genes *Acc1* and *Fas* in WAT by 46% and 35%, respectively, compared to the HF group. However, the low concentration of SL extract reduced the lipogenesis-related gene *Acc1* by 86% in the liver compared to the HF group (Figure 7D). As shown in Figure 7B, the high concentration of SL extract reduced adipogenesis-related genes *C/ebpa* (69%) and *Srebp1* (28%) in WAT compared to the HF group. The low concentration of SL extract reduced the adipogenesis-related gene *C/ebpa* in the liver by 86% (Figure 7E). Figure 7C,F show that the high concentration of SL extract increased the thermogenesis-related genes *Ampk* by 150%, *Ppara* by 40%, *Ucp1* by 76% in WAT, and *Ampk* by 965%, *Pgc1a* by 592%, *Ppara* by 300%, and *Ucp1* by 158% in the liver compared to the HF group. Furthermore, the high concentration of SL extract increased the ratio of pAMPK/AMPK and decreased p-ACC1/ACC1 compared to that in the HF group (Figure 7G–I), whereas the low concentration increased the UCP1 protein expression (Figure 7J). According to these results, SL extract can reduce the weight of mice fed a high-fat diet by inhibiting lipogenesis and adipogenesis and activating thermogenesis-related genes.

### 3.8. Effects of S. lomentaria Extract on the Gut Microbiota Composition in High-Fat-Fed Mice

**Figure 8:**
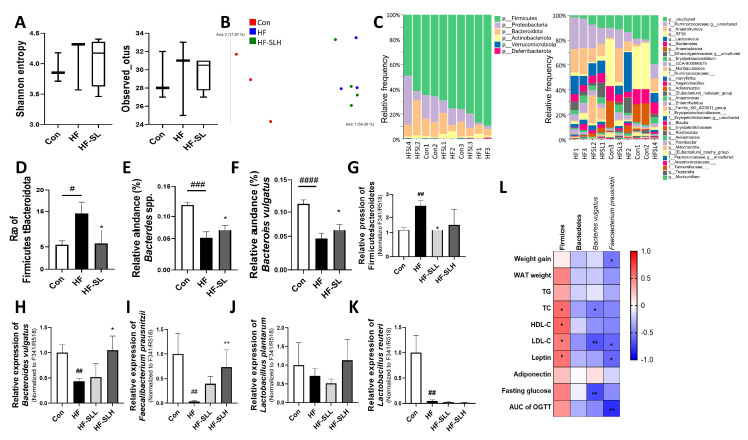
Effects of *S. lomentaria* (SL) extract on the gut microbiota in mice fed a high-fat diet. (**A**) Shannon and observed ASVs diversity. (**B**) PCA of unweighted UniFrac. (**C**) Taxonomy analysis of community at phylum and genus levels. (**D**) The ratio of Firmicutes to Bacteroidetes. (**E**) Relative abundance of Bacteroides spp. and *(***F**) *Bacteroides vulgatus* (*n* = 3–4/group; fecal DNA from two mice were pooled). (**G**–**K**) PCR results for specific bacteria (*n* = 10/group). The abundance of bacteria was quantified by total bacteria (F341/R518). The four diet groups: chow diet (Con), high-fat diet (HF), HF plus SL 150 mg/kg bw (HF-SLL), HF plus SL 300 mg/kg bw (HF-SLH). Data are expressed as mean ± standard deviation (*n* = 10). Compared with the HF group, significance was determined as # *p* < 0.05, ## *p* < 0.01, ### *p* < 0.001, #### *p* < 0.0001, Kruskal–Wallis test. Compared with the HF group, significance was determined as * *p* < 0.05, ** *p* < 0.01 using non-parametric Kruskal–Wallis test. (**L**) Analysis of the Spearman correlation between the abundance of gut microbiota and obesity-related markers (* *p* < 0.05, ** *p* < 0.01).

Dietary changes, particularly the consumption of high-fat diets, can influence the composition of the gut microbiota [32]; therefore, the effect of high doses of SL extract (300 mg/kg bw) on the fecal microbiota of obese mice were examined. As shown in Figure 8A, Shannon diversity and the observed operational taxonomic unit indices were not statistically significant in any of the groups. β-diversity differed between the con group and the HF and HF-SL groups, but not between the HF and the HF-SL groups (ANOSIM: Con vs. HF: R = 1 (*p* = 0.083); Con vs. HF-SLH: R = 1 (*p* = 0.028); HF vs. HF-SLH: R = 0.25 (*p* = 0.15); Figure 8B). In the 16S sequencing results (Figure 8C), as shown in Figure 8D, the ratio of Firmicutes to Bacteroidetes (F/B) was significantly higher in the HF group than in the Con group but decreased in the SL group relative to the HF group (*p* = 0.02). At the genus level, the populations of *Bacteroides* spp. were reduced by the high-fat diet and increased by the SL treatment (Figure 8E). Similar results were observed in the abundance of *Bacteroides vulgatus* (Figure 8F). When the 16S result was confirmed using qPCR, the F/B ratio was significantly higher in the HF group but decreased by the low concentration of SL treatment (Figure 8G). The abundance of *B. vulgatus* was reduced by a high-fat diet and was increased by the SL treatment (Figure 8H). When we measured the abundance of 10 well-known probiotics (listed in Supplemental Table S2) using qPCR, the abundance of most of them was not significantly different between the groups. However, the abundance of *Faecalibacterium prausnitzii* was only decreased in the HF group compared to the Con group and increased in the low concentration of SL treatment group compared to that in the HF group (Figure 8I). When examining the correlation between the gut microbiota distribution and obesity-related markers, Firmicutes displayed a positive correlation with TC, HDL-C, LDL-C, and leptin, whereas *B. vulgatus* demonstrated a negative correlation with TC, LDL-C, and fasting glucose. *F. prausnitzii* was also negatively correlated with weight gain, LDL-C, leptin, and OGTT results (Figure 8L). Therefore, it suggests that SL extract can improve obesity by improving the gut microbiota composition.

## 4. Discussion

Our study demonstrated that SL extracts have anti-obesity properties by reducing ACC1, C/EBPα, and the F/B ratio, and by inducing AMPK, UCP1, *B. vulgatus*, and *F. prausnitzii*. We confirmed our hypothesis that SL extract could alleviate obesity by suppressing lipogenesis and adipogenesis, enhancing thermogenesis, and changing gut microbiota.

Obesity is caused by an increase in the number of fat cells known as adipogenesis, and an increase in the size of individual fat cells known as lipogenesis [33,34]. Numerous in vitro and in vivo studies have identified multiple mechanisms for treating obesity, including balancing energy intake, reducing preadipocyte differentiation, fatty acid biosynthesis, cholesterol synthesis, and increasing thermogenesis [35]. Among the underlying mechanisms, AMPK is a key enzyme involved in the pathways of energy and lipid metabolism and has become a major target for treating obesity [36]. AMPK activation inhibits downstream targets involved in obesity [37], such as ACC1 and FAS, which are involved in lipogenesis [38], and PPARγ, C/EBPα, and SREBP-1C, which are involved in adipocyte differentiation and lipid accumulation [39]. Thermogenesis induction is essential for the suppression of obesity and mitochondrial biogenesis, through the activation of the thermogenic effector UCP1, and PGC1-α is a crucial thermogenesis driver [40]. Several natural polyphenols have been reported to exert anti-obesity effects by inhibiting adipocyte differentiation through the suppression of C/EBPα, PPAR-γ, FAS, SREBP-1C, and lipid metabolism (ACC-1, AMPK, FAS, and PPAR-α) [41]. Therefore, this study investigated the anti-obesity effect of polyphenol-rich SL extract by focusing on the following mechanisms: inhibition of adipogenesis and lipogenesis, and promotion of adipocyte browning.

Seaweed extracts are an excellent source of bioactive compounds that aid weight loss [42]. Catechins, fucoidans, and fucoxanthin have been identified in SL [12,43,44]. We also discovered sinapic acid, catechins, quercetin, hydroxytrifuhalol A, acacetin, and caffeic acid in SL extract. These ingredients have been demonstrated to inhibit lipogenesis and adipogenesis and increase thermogenesis. Sinapic acid exhibited anti-obesity effects in multiple cell studies by downregulating *Pparg*, *C/ebpa*, *Srebp-1c*, and *Fas* [45] and upregulating *Ucp1*, *Pgc1a*, and *Prdm16* [46,47]. Catechin and Quercetin reduced obesity by regulating lipid metabolism and stimulating non-shivering thermogenesis (*Ucp1*, *Pgc1a*) [48,49]. Acacetin has been reported to reduce body weight and adipose tissue weight by inhibiting adipogenesis-related transcription factors *C/ebpa* and *Srebp-1c*, by and enhancing the phosphorylation of AMPKα and ACC-1 [50,51]. SL extract contains these components and might potentially exert anti-obesity effects. Therefore, this study investigated the anti-obesity activities of SL extract in 3T3-L1 adipocytes and an obese mouse model.

3T3-L1 cell differentiation is a well-established model of adipogenesis with lipid accumulation. During adipocyte differentiation, a number of transcriptional cascades are activated. PPARγ and C/EBPα, which are involved in differentiation and govern adipogenesis at an earlier level [52,53], are the master regulators of adipogenesis. FAS, FABP4, and ACC1 are involved in lipid synthesis and participate in the late stages of differentiation [54,55]. In this study, SL extract reduced the formation of lipid droplets during 3T3-L1 cell differentiation. Additionally, SL extract decreased *C/ebpa*, *Pparg*, *Acc1* mRNA, and pACC1 protein while increasing *Ucp1* mRNA, UCP1, and pAMPK protein. The accumulation of lipid droplets in adipocytes causes obesity. The suppression of the master regulators of adipogenesis, *Pparg*, *C/ebpa*, and *Acc1* by SL extract is indicative of its potential anti-adipogenic and anti-obesity effects. In this mouse model fed a high-fat diet, SL extract lowered fat mass, body weight, and adipocyte size compared to the HF group. In epididymal fat tissue, lipogenesis- (*Acc1*, *Fas*) and adipogenesis (*C/ebpa*, *Srebp1*)-related genes were decreased in the HF-SLH group compared to the HF group, although thermogenesis-related *Ampk*, *Ppara*, and *Ucp1* genes were enhanced by SL extract. This reveals that SL extract can inhibit adipogenesis and lipogenesis similarly to 3T3-L1 cells. However, SL extract had minimal effects on adipogenesis and lipogenesis-related genes in the liver, and SL extract at a high dose only increased energy metabolism and thermogenesis-related genes *Ampk*, *Pgc1a*, *Ppara*, and *Ucp1* compared to the HF group. This is likely because the liver has not yet accumulated lipids, as evidenced by histological scoring, but SL extract still had the potential to promote energy metabolism in the liver compared to the HF group. Consequently, by inhibiting lipogenesis and adipogenesis and promoting thermogenesis, SL extract may help prevent obesity.

Treating hyperlipidemia and hyperglycemia has the potential to treat and prevent obesity [56]. In these animal experiments, eight weeks of high-fat diet significantly increased fat mass, body weight, HDL-C, LDL-C, TG, and TC levels, and the levels of fasting blood glucose and OGTT, resulting in hyperlipidemia and hyperglycemia. Compared to the HF group, SL extract treatment significantly decreased body weight, fat mass, oral glucose tolerance, fasting blood glucose, and LDL cholesterol. Leptin is an adipokine that regulates body metabolism and appetite [57], and obesity is associated with leptin dysregulation, which increases the risk of cardiovascular disease [58]. Administration of SL extract significantly decreased the leptin level, suggesting that SL extract may have a beneficial effect on obesity and cardiovascular risks. Previous studies demonstrated that sinapic acid, one of the components of SL extract, ameliorated hyperglycemia by inducing PLC/PKC signals to increase glucose utilization [59]. Consequently, it was observed that SL extract have the potential to reduce hyperlipidemia and hyperglycemia in obese mice.

Dysbiosis of the gut microbiota is intimately associated with obesity and promotes the genesis and progression of obesity by increasing appetite, chronic inflammation, and fat storage in the host [25]. Recent studies have investigated the role of natural compounds in modifying the gut microbiota to prevent obesity [16,60]. The F/B ratio is increased in obese mice and humans, resulting in a pro-inflammatory signature of the obese microbiota [61]. SL extract decreased the F/B ratio induced by a high-fat diet. Further, 47 species-level bacteria were identified by 16S sequencing, and qPCR analysis of 10 known probiotics was also performed. However, among them, the abundance of *F. prausnitzii* and *B. vulgatus* was only significantly decreased by the high-fat diet but increased by SL extract. *B. vulgatus*, belonging to the phylum Bacteroides, is the predominant gut microbial species depleted in obese individuals and plays a crucial role in maintaining a healthy gut ecosystem [59]. *F. prausnitzii* is a probiotic that generates butyrate, an anti-inflammatory molecule that regulates the intestinal immune system, oxidative stress, and metabolism of colon cells [62]. In this study, the abundance of Firmicutes was positively correlated with obesity-related markers, whereas *F. prausnitzii* and *B. vulgatus* were negatively correlated with a number of obesity-related markers. In addition, the abundance of *F. prausnitzii* and *B. vulgatus* was significantly decreased by the high-fat diet but increased by SL extract. Therefore, SL extract exhibited anti-obesity effects by modulating gut microbiota composition.

Prior to identifying the underlying mechanism of action, it is essential to establish the optimal dosage of bioactive chemicals. However, SL ethanol extract has only been tested on cells and zebra fish [12,14,15,17] and has not yet been trialed on animals. In order to assess the dosage of SL extract in our animal research, we employed *Ecklonia cava* ethanol extract (ECE), which has undergone extensive toxicity testing in conjunction with other brown algae. The NOAEL value for ECE is 750 mg/kg, which does not indicate toxicity [63]. According to a number of studies, a dose of 150 mg/kg ECE significantly reduces body weight and fat mass in mice fed a high-fat diet [64]. In other experiments, however, rats fed a high-fat diet and treated with ECE for eight weeks at dosages of 250 mg/kg and higher lost weight [65]. In a separate study, mice on a high-fat diet were treated with 300 mg/kg ECE for 10 weeks, which reduced body weight and improved hepatic steatosis [66]. This research revealed no evidence of toxicity in brown algae. Based on these studies, we selected to administer 150 and 300 mg/kg body weight of SL extract in this investigation. Nonetheless, additional research is required to determine the optimal dosage and toxicity of SL extract.

One of the experimental limitations was that that the analysis of the identified compounds in SL extract was a preliminary screening that requires additional experiments. Currently, we are in the process of establishing a library of components found in brown algae by conducting a literature review and estimating the substance by comparing the molecular weight and fragment pattern of the library based on the results of LC-MS analysis. Only six substances that could be clearly estimated have been identified in this study and the remaining peaks are still in the process of confirmation. On the basis of the peak area, the six substances were considered to be the primary components in SLE, as compared to other components, and to be most correlated with observed biological activity. However, additional confirmation is necessary. Another limitation was that this animal study lasted only eight weeks. There were no differences in liver weight among the groups, and there was no evidence of fat deposition in the liver. A recent study demonstrated that feeding mice an HF diet for 12 weeks induced hepatic steatosis [16], suggesting that we discontinued the study and terminated the experiment at Week 8 prior to the adipogenic phase. Therefore, to confirm the effects of bioactive compounds on adipogenesis in animal models, it is essential to feed mice a high-fat diet for a minimum of 12 weeks. The other drawback was the use of a chow diet as the control diet. The composition of the control diet should be identical to that of the HFD diets, with the exception of a lower fat percentage. In this study, however, a diet of commercial chow was utilized, which contained various types of protein and varied amounts of fiber, vitamins, and minerals compared to the HF diet. For future experiments, it would be essential to use a custom-formulated chow diet containing the same sources and proportions of nutrients as the HF diet.

## 5. Conclusions

This investigation revealed that the SL extract reduced weight gain and WAT weight, reduced LDL-C, leptin, and improved glucose intolerance. *Scytosiphon lomentaria* suppressed the expression of genes and proteins involved in adipogenesis, lipogenesis, and thermogenesis. In addition, treatment with SL extract ameliorated gut microbiota changes caused by a high-fat diet. Based on these changes, SL extract may have the potential to reduce obesity by altering adipogenesis, lipogenesis, thermogenesis, and gut microbiota composition, and SL extract can be used as anti-obesity agents.

## Figures and Tables

**Table 1:** Chemical characterization of *S. lomentaria* extract by LC-MS.

Peak	Compound Name	Molecular Formula	Retention Time (min)	[M+H]^+^	Fragment Ion (*m*/*z*)	Content *
1	Sinapic acid	C_11_H_12_O_5_	1.348	225.0757	208.1340, 207.0781, 206.0697	13.00
2	Catechin	C_15_H_14_O_6_	1.348	291.0863	207.0781, 140.0687, 122.0968	13.00
3	Quercetin	C_15_H_10_O_7_	1.348	303.0499	302.1982, 258.1327, 202.1808	13.00
4	Hydroxytrifuhalol A	C_18_H_14_O_11_	11.289	407.0609	407.2007, 391.2078, 361.1700, 249.1461	16.44
5	Acacetin	C_16_H_12_O_5_	12.381	285.0757	243.1123, 242.1096, 241.1044	1.99
6	Caffeic acid	C_9_H_8_O_4_	12.669	181.0495	182.1262, 180.1135, 181.1230,	5.33

* µg Quercetin/mg extract.

## Data Availability

The datasets generated during and/or analyzed during the current study are available from the corresponding author H.K. on reasonable request.

## Supplementary Materials

The following supporting information can be downloaded at: https://www.mdpi.com/article/10.3390/nu15040815/s1, Table S1. Sequences of primers used for PCR. Table S2. Sequences of primers used for bacterial profiling.
