# Supplementary material for: Scytosiphon lomentaria Extract Ameliorates Obesity and Modulates Gut Microbiota in High-Fat-Diet-Fed Mice
Source: Nutrients. 2023 Feb 5;15(4):815. doi: 10.3390/nu15040815 (PMC9965426; doi:10.3390/nu15040815)
Supplement: Supplementary file 1 [file nutrients-15-00815-s001.zip › nutrients-2170262-supplementary.pdf]

**Supplemental Table S1. Sequences of primers used for PCR.**

| Gene | Forward | Reverse |
|---|---|---|
| *β-actin* | CCCTACAGTGCTGTGGGTTT | GAGACATGCAAGGAGTGCAA |
| *Acc1* | GCCTCTTCCTGACAAACGAG | TGACTGCCGAAACATCTCTG |
| *Ampk* | TGTTCCAGCAGATCCTTTCC | ATAATTGGGTGAGCCACAGC |
| *C/ebpa* | ATCAGCGCCTACATTGATCC | TTGCTTGGCTGTCGTAGATG |
| *Fas* | CCCTTGATGAAGAGGGATCA | ACTCCACAGGTGGGAACAAG |
| *Pgc1a* | AATGCAGCGGTCTTAGCACT | GTGTGAGGAGGGTCATCGTT |
| *Ppara* | TCTTCACGATGCTGTCCTCCT | CTATGTTTAGAAGGCCAGGC |
| *Pparg* | CCCTGGCAAAGCATTTGTAT | GAAACTGGCACCCTTGAAAA |
| *Prdm16* | GGCATGACTTGCTGACTCAA | GGTGTGGACTGGTCTGGTCT |
| *Srebp1* | GAGCCTTCAGACACGTCCTC | ACTCTTCTGGTGTGGCTGCT |
| *Ucp1* | CTGCCAGGACAGTACCCAAG | GCCACAAACCCTTTGAAAAA |

**Supplemental Table S2. Sequences of primers used for bacterial profiling**

| Target | Forward | Reverse | Reference |
|---|---|---|---|
| Uni (F341/R518) | CCTACGGGAGGCAGCAGT | ATTACCGCGGCTGCTGG | Lubbs (2009) |
| Bacteroidetes | GGARCATGTGGTTTAATTCGATGAT | AGCTGACGACAACCATGCAG | Guo (2008) |
| Firmicutes | TGAAACTYAAAGGAATTGACG | ACCATGCACCACCTGTC | De Gregoris (2011) |
| *Akkermansia muciniphila* | CTGAACCAGCCAAGTAGCG | CCGCAAACTTTCACAACTGACTTA | Collado (2007) |
| *Bifidobacterium bifidum* | ATTTGAGCCACTGTCTGGTG | CATCCGGGAACGTCGGGA AA | Sul (2007) |
| *Bifidobacterium breve* | AATGCCGGATGCTCCATCACAC | GCCTTGCTCCCTAACAAAAGAGG | Rinne (2005) |
| *Bifidobacterium longum* | GGATGTTCCAGTTGATCGCATGGTC | AGCCGGTGCTTATTCAACGGGTAA | Bergstrom (2012) |
| *Bacteroides vulgatus* | GCATCATGAGTCCGCATGTTC | TCCATACCCGACTTTATTCCTT | Wang (1996) |
| *Clostridium butyricum* | GTGCCGCCGCTAACGCATTAAGTAT | ACCATGCACCACCTGTCTTCCTGCC | Bartosch (2004) |
| *Faecalibacterium prausnitzii* | AGATGGCCTCGCGTCCGA | CCGAAGACCTTCTTCCTCC | Wang (1996) |
| *Lactobacillus plantarum* | AATTGAGGCAGCTGGCCA | GATTACGGGAGTCCAAGC | Quere, 1997 |
| *Lactobacillus reuteri* | GCCGCCTAAGGTGGGACAGAT | AACACTCAAGGATTGTCTGA | Walter (2000) |
| *Lactococcus lactis* | TGAAGAATTGATGGAACTCG | CATTGTGGTTCACCGTTC | Bachmann (2015) |
